# Nipple or skin-sparing mastectomy flap: can preoperative breast-MRI predict post-operative complications?

**DOI:** 10.1007/s13304-026-02843-z

**Published:** 2026-10-13

**Authors:** L. I. Sgaramella, A. Pasculli, F. P. Prete, F. Cofone, M. Moschetta, M. Maruccia, R. Elia, M. Telegrafo, V. De Ruvo, M. Bisceglie, M. M. Miccoli, F. Palma, C. Testini, G. Giudice, M. Testini, A. Gurrado, BCUPoliBa Working Group, C. Angelini, C. Caporusso, M. E. Caringella, C. Deleo, F. Iuele, T. Grassi, E. Lacalendola, M. Lambo, W. Lavermicocca, V. Lorusso, A. Nitti, M. Piombino, A. R. Pisani, E. Poli, F. Puntillo, N. Resta, G. Rubini, M. Rugge, G. Serio, F. Solfrizzo, L. S. Stucci

**Affiliations:** 1 Department of Precision and Regenerative Medicine and Ionian Area (DiMePreJ), Aldo Moro University, Bari, Italy; 2 Breast Care Unit, Policlinico of Bari, Bari, Italy; 3 Diagnostic Senology Unit, AUOC, Policlinico of Bari, Bari, Italy; 4 Department of Interdisciplinary Medicine (DIM), University of Bari Aldo Moro, Bari, Italy; 5 Pathology Unit, AUOC, Policlinico of Bari, Bari, Italy; 6 Radiation Oncology Department, AUOC, Policlinico of Bari, Bari, Italy; 7 Medical Oncology Unit, AUOC, Policlinico of Bari, Bari, Italy

**Keywords:** Breast cancer, Skin sparing mastectomy, Nipple-sparing mastectomy, Breast magnetic resonance imaging, Skin flap thickness, Postoperative complications, Breast reconstruction

## Abstract

**Background:**

This study aimed to evaluate the role of skin flap thickness (SFT), measured by preoperative breast magnetic resonance imaging (MRI), in predicting postoperative complications after nipple-sparing or skin-sparing mastectomy, and to assess its value in guiding reconstructive strategy selection.

**Methods:**

We conducted a retrospective single-center study including patients who underwent preoperative breast MRI and nipple-sparing or skin-sparing mastectomy for breast cancer or BRCA1/2 mutations between January 2019 and August 2025. Demographic, clinical, and perioperative data were collected. Patients were stratified into two groups based on mean preoperative SFT: >8 mm (Group A) and <8 mm (Group B). Postoperative complications were recorded and analyzed using univariate and multivariate models.

**Results:**

Sixty patients (mean age 47.5 ± 9.1 years) and 80 breasts (40 unilateral, 20 bilateral procedures) were included. Group A comprised 54 breasts (67.5%), while Group B included 26 (32.5%). Postoperative hospital stays and incision type differed significantly between groups (*p* < 0.05). Overall postoperative morbidity was 16.25%. Partial nipple–areola complex necrosis (11.5%) and hematoma (7.7%) occurred significantly more often in Group B compared to Group A (both *p* < 0.05). Multivariate analysis confirmed preoperative SFT <8 mm as an independent predictor of these complications (odds ratio 13.40, *p* = 0.012).

**Conclusion:**

Preoperative magnetic resonance imaging assessment of skin flap thickness reliably identifies patients at increased risk of postoperative complications. Integrating this parameter into surgical planning may support personalized reconstructive decision-making and improve clinical outcomes.

## Introduction

Breast cancer (BC) is the leading cause of cancer morbidity, disability and mortality in women worldwide. In Europe the estimated incidence is 147.6 per 100,00 population, while in 2023 in Italy is 55.900, with a 5-year survival rate of 88% [1, 2].

During the last decades, advances in early-stage detection and targeted therapies have led to a considerable improvement in BC therapy and overall survival rates [3]. In this context, nipple-sparing or skin-sparing mastectomy (NSM/SSM) have become the cornerstone of surgical and oncological management of BC. By preserving the native skin envelope and nipple areolar complex (NAC), when possible, these techniques have optimized aesthetic outcomes, improving body image and patients’ satisfaction without compromising oncological outcomes [4–7].

Currently, breast reconstruction techniques can be divided into autologous reconstruction with flaps [transverse rectus abdominis (TRAM), deep inferior epigastric perforators (DIEP), and others] and heterologous reconstruction (prosthesis-based techniques), preferred in about 80% of mastectomies [8, 9].

Implant-based reconstruction can be one-stage (a single surgical procedure with the implant is usually enclosed by an acellular dermal matrix, in a prepectoral pocket) or two-stage (with the placement of an expander in the retro-pectoral position during the mastectomy, followed by the implantation of the prosthesis later) [8].

According to current guidelines an immediate reconstruction is indicated for patients with a body mass index (BMI) between 25 and 30 able to guarantee a good vascularization of skin flap [10–12].

The preservation of blood supply of the envelopment tissue in NSM/SSM is, indeed, crucial in ensuring good aesthetic and reconstructive outcomes [13]. It is well known that this technique can be more technically challenging than breast conservative surgery and may carry unique complication risks with an overall complication rate ranging from 20 to 30% [14–16].

Technique-related complications can be distinguished in bleeding, infection, thrombosis/necrosis of the flap, skin breakdown/wound complication, seroma, implant loss, or nipple necrosis/ischemia/eschar and may negatively affect patient-reported outcomes [14, 15]. Although the importance of flap thickness and anatomical dissection during mastectomy has been widely emphasized, a standardized method for the objective preoperative assessment of skin and subcutaneous tissue thickness in patients undergoing SSM and NSM has not yet been established. Nevertheless, recent studies have identified a flap thickness of approximately 8 mm as a relevant reference threshold for surgical planning and outcome evaluation [17].

The aim of this retrospective cohort study is to compare the skin flap thickness < or > 8 mm at MRI in terms of early post-operative complications after SSM/NSM for BC or BRCA 1/2 mutation.

## Materials and methods

We conducted a single-center, retrospective observational study to investigate whether the integration of demographic and preoperative magnetic resonance imaging (MRI) data can predict postoperative complications in a population of patients undergoing SSM or NSM with one- or two-stage breast reconstruction, performed either for BC treatment or prophylactically due to BRCA1/2 mutations.

The study received approval from the Independent Ethical Committee of the University of Bari (Protocol 1745/CEL, 03/07/2024) and was performed in accordance with the Declaration of Helsinki of 1975, revised in 2013.

All patients affected by BC or BRCA 1/2 mutation preoperatively studied with breast-MRI and submitted to SSM/NSM followed by one- or two-stage breast reconstruction were identified from the prospective database of our Academic Unit of Surgery from January 2019 to August 2025 to be enrolled in the study.

After diagnosis of BC or BRCA 1/2 mutation, all patients were followed up by the multidisciplinary team (Breast Care Unit of Policlinic of Bari – BCU). The most appropriate therapeutic strategy was established in accordance with recent guidelines [10–12].

All patients provided informed consent to treatment and study participation. Patients were divided in 2 groups (A and B) according to mean preoperative MRI thickness< or > 8 mm. The present study is reported in line with the STROBE guidelines [18]

### Patient selection

Inclusion criteria for the study were the following: gender female, age >18, pre-operative breast-MRI, breast weight and implant size, NSM/SSM with subsequent two-stage reconstruction with expander or one-stage reconstruction with dermal matrix prosthesis implantation. History of surgery in the same breast or previous irradiation of the chest wall were not cause of exclusion.

Exclusion criteria were stage IV disease; breast conserving surgery (BCS); mastectomy without reconstruction or reconstruction with autologous tissue; unavailability of MRI; refusal to participate to study or complete follow-up.

### Surgical technique

All mastectomies were performed, under general anesthesia, by the same experienced surgeons (AG, MM).

SSM or NSM were performed through skin incision depending on breast size and the distance between the inframammary fold and the clavicle. For patients with large and/or ptotic breasts undergoing NSM, a Wise-pattern skin-reducing incision was adopted. In these cases, the NAC was preserved on an inferior dermal pedicle whenever oncologically feasible, allowing reduction of the skin envelope while maintaining the vascular supply to the NAC. Free nipple grafting was not performed in any patient included in this study. In SSM, NAC and all glandular tissue were removed preserving the subcutaneous fat, which was checked to be sufficiently thick to maintain the vascular plexus for the skin and subcutaneous tissue. NSM is closely similar except that NAC is also conserved when the fresh frozen retroareolar biopsy was negative. In all cases, before reconstruction, the breast weight was measured in grams.

No intraoperative perfusion imaging techniques (e.g., indocyanine green angiography) were routinely employed. Flap viability was assessed by clinical evaluation based on color, capillary refill, dermal bleeding, and tissue appearance, while flap thickness was estimated intraoperatively by the operating surgeon.

In one-stage reconstruction cases, if the subcutaneous plexus and the cutaneous flap were adequately thick, a prosthetic implant (MENTOR CPG™ Gel Breast Implants with Cohesive III™, Mentor, Santa Barbara, CA, USA) was inserted, covered by an acellular dermal matrix, pre-shaped, of porcine origin, non-cross-linked (ADM Braxon® (MBP Biologics, Neustadt-Glewe, Germany, license holder Decomed, Marcon, Venice, Italy)), into the prepectoral pocket, fixed to the pectoralis major muscle with absorbable sutures.

In the two-stage reconstruction, a tissue expander (MENTOR CPX™4 Breast Tissue Expander, Mentor, Santa Barbara, CA, USA) was placed in the submuscular pocket, created by suturing the pectoralis major muscle to the serratus anterior. The size of the expander was selected based on the diameter and volume of the pocket.

The expander was filled with approximately 50–100 cc of saline solution and 2 cc of methylene blue and was expanded every 1–2 weeks until the desired final volume was reached. After 4–6 weeks from the final expansion or upon completion of adjuvant therapy, the expander was replaced with a permanent implant.

### Data collection and outcome evaluation

Patients’ demographic data including general characteristics, comorbidity and medications, pre-operative diagnosis, neoadjuvant therapies (NACT), history of previous breast surgery and/or radiotherapy, side of BC and pre-operative MRI data were retrieved from our prospective database. Data on operative time, type of incision, of mastectomy and reconstruction, mastectomy flap thickness, breast weight, implant size, hospital stay, and duration of drain stay were also recorded.

Minimum follow-up was 3 months across the whole study population. The primary endpoint was to evaluate the association between preoperative MRI-derived mastectomy skin flap thickness and the occurrence of any early postoperative complication within three months after surgery. Secondary endpoints included the association between flap thickness and each specific postoperative complication as well as the identification of independent predictors of postoperative complications through multivariable analysis.

All early perioperative complications (including surgical site infection –SSI-), flap or NAC necrosis, wound dehiscence, implant loss, red breast syndrome (RBS), seroma, edema, and hematoma/bleeding) were ranked according to the Clavien–Dindo classification and noted along with the relative management strategies (including reoperation and readmission) [19].

SSI was defined as an infection related to an operative procedure that occurs at or near the surgical incision within 30 days of the procedure or within 90 days if prosthetic material is used and implanted [20].

Breast oedema was specified by a post-operative ultrasound presentation of skin thickening, increased parenchymal echogenicity and interstitial marking. Seroma was defined as the accumulation of serous fluid (volume of more than 5 to 20 ml of fluid) with clinical evidence of palpable, fluctuant, ballotable, or tense region. It could require at least one needle aspiration of inflammatory exudate [21].

A breast hematoma is a collection of blood that accumulates in the breast tissue, presenting with an area of blue/ yellow color of the skin and subcutaneous fat [22].

RBS is an inflammatory event that typically presents with cutaneous erythema over the domain where the acellular dermal matrix is surgically implanted [23].

Ischemic complications were defined as partial or total full-thickness necrosis of the flap or of the NAC. Wound dehiscence was defined as the widening of the surgical wound, while implant loss as the visibility of the implant through the skin needing for a second surgery to revise the tissue expander or prosthesis [22, 23].

### MRI evaluation

All data regarding preoperative MRI were analyzed by breast imaging specialists (MM, FC). As blinded reviewers, they measured flap thickness on preoperative MRI (skin-to-gland flap) using PACS software on sagittal and axial images acquired using a dedicated breast coil on 1.5 Tesla magnets with T2-weighted volumetric scans.

**Fig. 1:**
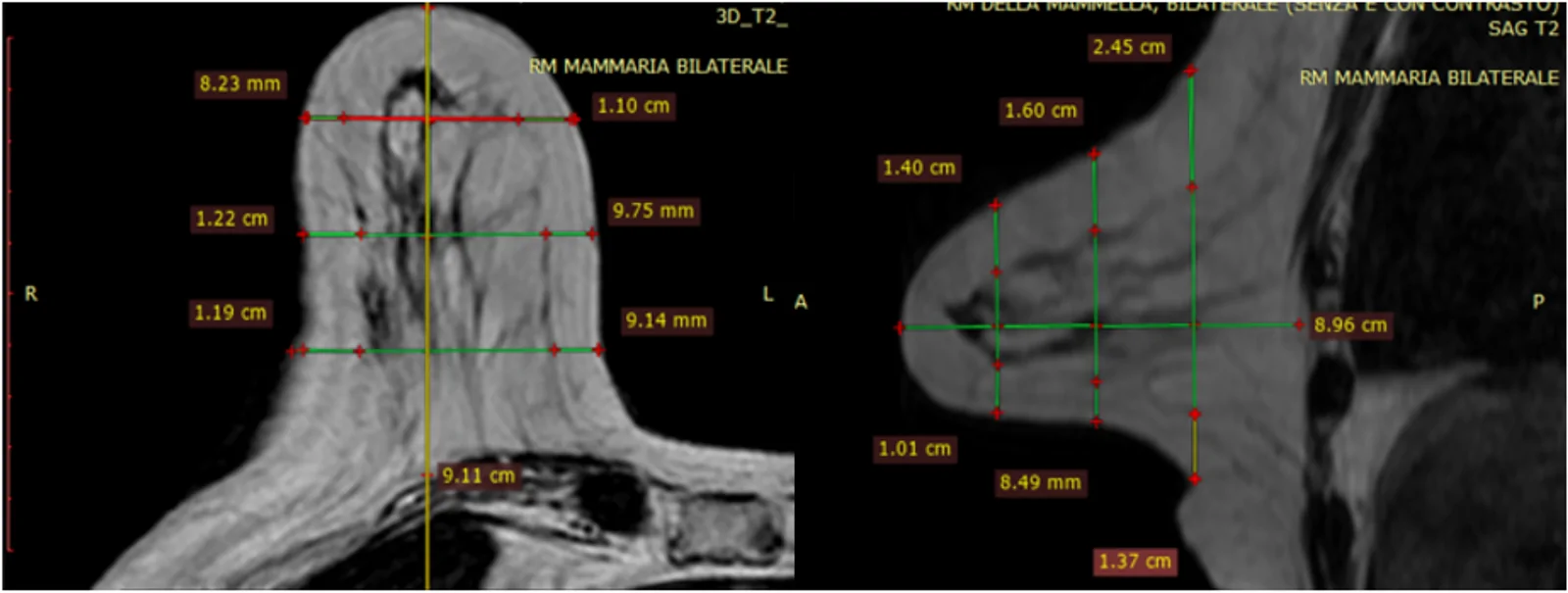
Preoperative MRI and skin flap thicknesses evaluation in different positions

On average, the thicknesses were calculated in 12 different positions (Fig. 1). Three measurements were taken on the upper and lower flaps, in the sagittal plane, and on the medial and lateral flaps in the axial plane. Finally, 3 measurements corresponding to the anterior, middle and posterior thickness were considered, taken at one-quarter, one-half and three-quarters of the length of the total anteroposterior diameter of each breast.

### Statistical analysis

Categorical variables were presented as number of cases/total (%), whereas continuous variables were presented as mean± standard deviation. The analysis of outcomes with respect to clinicopathological and perioperative parameters was performed based on the glands that were subjected to the intervention. For the purposes of analysis, the population study was then divided into 2 groups based on mean preoperative MRI thickness: group A > 8 mm and group B < 8 mm.

The Kruskal–Wallis ANOVA test was used to compare differences in continuous variables between groups. Descriptive statistics and summary values were analyzed with Student’s t-test for independent samples for continuous values or χ2/Fisher’s exact test for ordinal/binomial variables. Multivariate analysis was performed using exact logistic regression, to relate significantly different outcomes with a model including mean thickness value along with the significant variables identified in the univariate analysis, reporting the Odds Ratio (OR) and 95% confidence intervals (95% CI) as factors for potential complications in breast reconstruction. Given the limited number of outcome events, the multivariable analysis was considered exploratory. To reduce the risk of model overfitting, only variables that reached statistical significance in the univariate analysis were included in the exact logistic regression model. The statistical analysis was conducted using SPSS® version 22.0.0 software (IBM, Armonk, New York, USA), and statistical significance was defined as *p* < 0.05.

## Results

From January 2019 to August 2025, 1468 patients underwent breast surgery at our Academic Unit of General Surgery. According to exclusion criteria, 1408 (95.9%) patients were not included in the study. Of these 1408 patients, 29 (2.06%) had stage IV disease, BCS 563 (40%), mastectomies without reconstruction 181 (12.8%), mastectomies with DIEP flap reconstruction 91 (6.5%), patients with unavailability of MRI 524 (37.2%), refusal to participate to study or complete follow-up 20 (1.4%).

The whole sample analyzed includes 60 patients (the mean age was 47.50 ± 9.10; range 29 to 67) and, within this sample, the operated breasts were 80 (40 unilateral mastectomy and 20 for bilateral mastectomy).

Analysis moved then to consider subgroups defined by mean preoperative MRI thickness: group A > 8 mm (n =54, 67.50%) and group B < 8 mm (n=26; 32.50%).

Table 1 shows demographic data, clinical characteristics, perioperative data and type of surgery.

**Table 1:** Demographic data, clinical characteristics, type of surgery, and intraoperative evaluation

	Group A (N = 54)	Group B (N = 26)	*p-value
Age	49.64 ± 9.10	43.30 ± 7.41	0.214
Mean duration of drain	13.98 ± 1.83	13.84 ± 1.93	0.420
Duration of surgery	105.45 ± 40	92.88 ± 40	0.618
Length of post operative hospitalization	52.78 ± 13.69	48.61 ± 11.35	**0.010**
BMI > 30	25 (46.3%)	8 (30.8%)	0.186
Smoking habits	4 (7.4%)	1 (3.8%)	0.538
Cardiologicaldisease	3 (11.5%)	3 (5.6%)	0.341
Respiratory. disease	2 (3.7%)	0	0.320
Connectivetissuedisease	6 (11.1%)	1 (3.8%)	0.281
Corticosteroid or immunosuppressantdrugs	2 (3.7%)	2 (7.7%)	0.443
Anticoagulant/antiplateletdrugs	0	1 (3.8%)	0.147
Previousbreast surgery	8 (14.8%)	3 (11.5%)	0.690
Previous neck/chestradiotherapy	3 (5.6%)	0	0.221
NACT	5 (9.3%)	3 (3.8%)	0.750
ASA > 3	0	0	-
Clinical intraoperative flap thickness evaluation	54 (100%)	26 (100%)	-
*Type of surgery*			
Skin sparing mastectomy	21 (38.8%)	6 (23.1%)	0.161
Nipple sparing mastectomy	33 (61.1%)	20 (76.9%)	
*Different skin incision*			
Wise pattern	18 (33.3%)	2 (7.7%)	**<0.001**
Periareolar	34 (63.0%)	14 (54.0%)	
Inframammary		2 (3.7%)	10 (38.5%)
*Reconstruction*			
One-stage	33 (61.1%)	11 (42.3%)	0.113
Two-stage	21 (38.9%)	15 (57.7%)	

Group A: mean preoperative MRI thickness > 8 mm. Group B: mean preoperative MRI thickness < 8 mm. ASA: American Society of Anesthesiologists. BMI: Body mass index. NACT: neoadjuvant chemotherapy

Data shown as average ± standard deviation or as absolute frequency with percentage in brackets. *Student’s t test for independent samples for continuous values or χ2/Fisher’s exact test for ordinal/binomial variables. Statistically significant values are shown in bold. Percentages are expressed for each group

Among the overall preoperative parameters evaluated, 33 patients had BMI >30 (55%), 6 patients had cardiological comorbidities (10%), 2 patient had pulmonary disease (3.33%), 7 patients had connective tissue disease (11.67%), no patients had ASA score > 3. No cases of alcohol abuse or drug use were recorded, and 5 patients had smoking habits (8.33%). Corticosteroids or immunosuppressant drugs and anticoagulants were reported in 4 (6.67%) and 1(1.67%) case, respectively, while previous NACT was reported in 8 cases (13.3%). Previous breast surgery was reported in 11 patients (18.34%), while surgical site irradiation was present in 3 cases (5%).

SSM was performed in 27 cases, while NSM in 53, through different skin incisions: 12 inframammary, 48 periareolar, 20 Wise patterns.

Relative to 80 breasts operated, in the 44 cases (55%) a one-stage, and in 36 (45%) a two-stage reconstruction was performed.

The mean breast weight was 374.8 ± 90.31 and in the prepectoral reconstruction cases, the mean of implant size was 382.16 ± 89.65.

The mean duration of surgery was 108 ± 40.72 min (range: 40–180), and length of post-operative hospitalization was 54 ± 13.13 h (range 36–72). The mean duration of drain stay was 14.06 ± 1.86 days (range 11–17).

No statistical differences were found between the groups, except for length of postoperative hospitalization (52.78±13.69 h) and type of the skin incisions (Wise pattern 33.3%, periareolar 63.0%) that were higher in group A than B (48.61 ± 11.35 h, 7.5%, 54.0%, respectively; all *P*<0.05). Instead, the inframammary incision was more frequent in B vs A (38.5% vs 3.7%, *P*<0.05).

Among the three cases of partial NAC necrosis, two occurred following NSM performed through a Wise-pattern skin-reducing incision, whereas one occurred in periareolar incision. No cases of free nipple grafting were performed in this series. Given the limited number of events, no statistically meaningful comparison according to incision type could be performed.

There was no perioperative mortality. Overall post-operative morbidity was 16.25%, while postoperative complications in two groups are detailed shown in Table 2.

**Table 2:** Post-operative complications

	Group A (N = 54)	Group B (N = 26)	*p-value
SSI	0	0	
Nipple areola necrosis			
partial	0	3 (11.5%)	**0.011**
complete	0	0	
Skin Flap necrosis			
partial	1 (1.9%)	2 (7.7%)	0.186
complete	0	0	
Wound dehiscence	1 (1.9%)	1 (3.8%)	0.593
Implantloss	1 (1.9%)	1 (3.8%)	0.593
Red breast syndrome	0	0	
Hematoma	0	2 (7.7%)	**0.039**
Seroma	1 (1.9%)	0	0.485
Re-surgery	1 (1.9%)	1 (3.8%)	0.593

Group A: mean preoperative MRI thickness > 8 mm. Group B: mean preoperative MRI thickness < 8 mm. SSI: surgical site infection. Data shown as average ±standard deviation or as absolute frequency with percentage in brackets. *Student’s t test for independent samples for continuous values or χ2/Fisher’s exact test for ordinal/binomial variables. Statistically significant values are shown in boldPercentages are expressed for each group

No statistical differences were found between the groups, except for partial NAC necrosis (11.5%) and hematoma (7.7%), that were more frequent in group B vs A (both 0%; all *P<*0.05).

The Table 3 shows the comparison between the significant complication group (partial NAC necrosis and hematoma; Group C, N=5) with no significant complication group (Group D, N= 75). The mean preoperative MRI thickness < 8 mm and comorbidities were significantly more frequent in group C (100% and 60%, respectively) vs D (28% and 13%, respectively; all *P<*0.05).

**Table 3:** Univariate analysis of partial nipple necrosis and haematoma risk factors

	Group C (N = 5)	Group D (N = 75)	P*
Age>40	5 (100)	63 (84)	1.000
BMI>30	2 (40)	31 (41)	1.000
ASA>3	0 (0)	0 (0)	n.a
Comorbidities	3 (60)	10 (13)	**0.028**
Smoke habits	0 (0)	5 (7)	1.000
Previous breast surgery	0 (0)	11 (15)	1.000
Previous neck/chest radiotherapy	0 (0)	3 (4)	1.000
NACT	1 (20)	7 (9)	0.418
Mean preoperative MRI thickness < 8 mm	5 (100)	21 (28)	**0.003**
Different skin incision	0.529		
Wise pattern	2 (40)	18 (24)	
Periareolar	2 (40)	46 (61)	
Inframmary	1 (20)	11 (15)	

Group C: significant complication group (partial NAC necrosis and hematoma); Group D: no-significant complication group. BMI: Body mass index. ASA: American Society of Anesthesiologists. NACT: neoadjuvant chemotherapy. MRI: magnetic resonance imaging. Data are expressed as absolute values and frequencies in brackets. *Fisher’s exact test for independent samples. Statistically significant values are shown in boldPercentages are expressed for each group

A multivariate analysis with exact logistic regression confirmed the significance of the differences between group C and D only for what concerns mean thickness value < 8 mm (*P<*0.05) (Table 4).

**Table 4:** Multivariate analysis of partial nipple necrosis and haematoma risk factors

	P*	OR (95% CI)
Comorbidities	0.125	7.97 (0.64–136.70)
Mean preoperative MRI thickness < 8 mm	**0.012**	13.40 (1.68-+∞)

MRI: magnetic resonance imaging. *Exact logistic regression

## Discussion

The objective of the study is to evaluate the impact of MRI skin flap thickness data in predicting postoperative complications after NSM/SSM for BC and BRCA 1–2 mutations.

Surgery still represents the most important therapeutic approach in localized BC, enabling eradication of macroscopic disease. The mastectomy should be considered for widespread ductal carcinoma in situ (DCIS), invasive breast cancer or in male patients.

The transition from total mastectomy to SSM and NSM in recent years has led to improved aesthetic and reconstructive outcomes and postoperative patient satisfaction, without compromising oncological safety [24, 25].

These surgical techniques were firstly described by Freeman in 1962 and modified by Toth and Lappert in 1991 [26, 27].

The underlying concept is to establish a dissection plane between the superficial layers and the glandular tissue, to allow complete gland removal while preserving the overlying flap and its vascularization.

Breast reconstruction following mastectomy can be performed using different approaches. Recent meta-analyses comparing prepectoral and subpectoral implant-based reconstruction demonstrated comparable rates of overall postoperative complications and reconstructive failure, while prepectoral reconstruction was associated with lower postoperative pain and absence of animation deformity in appropriately selected patients. These findings further emphasize that accurate preoperative patient selection remains the cornerstone of successful implant-based breast reconstruction [28, 29].

Immediate reconstruction may involve direct-to-implant placement, typically in the prepectoral plane with or without the support of an acellular dermal matrix (ADM), whereas staged reconstruction commonly consists of tissue expander placement followed by implant exchange. However, these do not represent the only reconstructive options currently available. Alternative approaches include prepectoral polyurethane-coated implants, prepectoral tissue expanders, and tissue expanders combined with ADM or other biological and synthetic meshes. Each technique has specific advantages and limitations and should be tailored to patient characteristics, oncologic factors, and mastectomy flap quality. One-stage reconstruction eliminates the need for a second procedure, reduces anesthesia exposure and hospitalization time, and may facilitate faster psychological and physical recovery [30, 31].

Conversely, two-stage reconstruction allows gradual expansion and volume modulation, potentially facilitating the management of wound-healing issues and soft-tissue adaptation. Given the technical demands of implant-based breast reconstruction and the critical importance of preserving adequate mastectomy flap vascularization and thickness, these procedures can be considered more complex than many other breast surgical operations, despite their well-established oncologic safety [32–35].

Dissection in an unidentifiable cleavage plane, indeed, may incur in the risk of incomplete oncologic resection in case of residual breast tissue, whereas aggressive dissection may excessively thin the flap, increasing complication risk due to impaired vascularization [17].

In SSM/NSM, the skin flap thickness has been widely discussed and, consequently, the optimal flap thickness has been the subject of extensive debate. A common recommendation has been to dissect superficially to the superficial fascia although its existence is not universally acknowledged. Beer et al. assessed the potential presence of the superficial fascia in histopathological samples, demonstrating that the superficial fascia was absent in 44% of breasts, limiting its reliability as a dissection plane [36].

These anatomical findings are consistent with the comprehensive description of the superficial fascial system provided by Rehnke et al., who demonstrated the complexity and variability of the breast fascial anatomy, highlighting why preservation of an adequate subcutaneous layer is crucial to maintain flap vascularity during mastectomy [37].

At the same time, Larson et al. demonstrated in histopathological samples the presence of a distinct and consistent layer of non-mammary subcutaneous tissue between the dermis and the mammary parenchyma, with a median thickness of approximately 1 cm, suggested as a safe oncologic limit ensuring flap viability [38].

Wiberg et al. reported a six-fold higher risk of postoperative necrosis in flaps ≤ 5 mm thick [39]

Rancati et al. proposed a preoperative patient-selection algorithm based on digital mammography, advocating preservation of mastectomy flaps thicker than 1 cm and ideally greater than 1.5 cm whenever oncologically feasible, in order to maintain not only the dermal and subdermal vascular plexus but also the subcutaneous vascular network [40].

The Rancati score has subsequently become one of the most widely adopted clinical tools for selecting candidates for immediate prepectoral implant-based breast reconstruction [40]. By integrating patient, breast, and treatment-related factors, it supports surgical decision-making aimed at reducing ischemic complications. However, although clinically useful, the score relies predominantly on clinical and mammographic parameters and does not include an objective quantitative assessment of mastectomy skin flap thickness. In this context, our findings suggest that preoperative MRI-derived flap thickness may provide complementary anatomical information, potentially improving patient selection, particularly in borderline cases in which the reconstructive strategy is uncertain.

Conversely, Frey et al. investigated the role of MRI in predicting mastectomy flap viability and identified a flap thickness below 8 mm as an independent predictor of ischemic complications [17].

The Author emphasizes also that, due to the difficulty of establishing flap thickness intraoperatively, tools such as MRI, which can provide a preoperative assessment of flap vitality and the ideal thickness, could be useful in preoperative assessment and in planning the type of reconstruction [17].

Therefore, while previous anatomical and reconstructive studies suggested that thicker flaps (> 1–1.5 cm) may represent the optimal condition, Frey et al. introduced the concept that a lower threshold of 8 mm could still be clinically relevant in identifying patients at increased risk of complications[17].

Based on these observations, in our study, postoperative outcomes after SSM/NSM are compared between patients who had skin flap thickness > or < 8 mm at preoperative MRI.

No significative difference was found between the two groups in terms of demographic and peri-operative features, except for length of hospitalization and different skin incisions.

In this regard, it is noteworthy that, in the absence of current guidelines on incision methods, various approaches are employed depending on breast size, tumor location, and surgeon preference [41].

In our study, the Wise pattern incision was statistically more frequently used in the group with a skin flap thickness > 8 mm, whereas the inframammary incision was more common in patients with a skin flap thickness < 8 mm. These findings thus support the current trend of preferring the Wise pattern incision for larger breasts, which are more likely to have greater tissue thickness, while the inframammary incision is typical for smaller glands, often associated with a thinner fat layer.

SSM/NSM could expose patients to the risk of procedure-related complications which can compromise the surgical outcome, threaten the entire reconstruction, delaying adjuvant therapies.

Among specific complications, patients could experience ischemic and hemorrhagic complications of flap and NAC that may impact reconstruction, with issues ranging from temporary wound complications to complete implant loss [40].

Recent literature reports a highly variable rate of ischemic complications. This variability appears to be related, among other factors, to the lack of a universally accepted definition. Indeed, some studies report ischemic complication rates on a per-patient basis, while others report them per-breast. Moreover, there is no unanimous consensus regarding the timing of onset, the extent of tissue involvement, or the criteria used to distinguish partial from total necrosis [42, 43].

Studies reporting mastectomy skin-flap necrosis describe an overall incidence ranging from approximately 2% to over 40%, although most series report average rates around 10%. Only a limited number of studies further stratify skin-flap necrosis into partial and complete forms, reporting rates of approximately 60.8% and 16.7%, respectively [42, 44]. NAC necrosis following NSM has been reported up to 20% across published series, with partial NAC necrosis rates of approximately 5.5% and complete NAC necrosis rates of approximately 3.5% [45].

Our study, however, reports an overall rate of 3.75% each for partial necrosis of NAC and for skin flap, and no cases of complete necrosis, although only the difference in terms of partial nipple areola necrosis is statistically significant.

In a meta-analysis evaluating outcomes in patients undergoing NSM with immediate implant-based breast reconstruction, the overall rate of ischemic complications or tissue necrosis was 9%, decreasing to 4% when specifically considering NAC necrosis or ischemia. The reported infection rate was 12%, while seroma formation occurred in 5% of cases. The explantation of implant was required in 4% of patients, and 9% experienced an unplanned return to the operating room. Notably, postoperative hematoma was reported in only 1% of cases [46].

By contrast, larger clinical studies including patients undergoing mastectomy with or without immediate reconstruction have reported a higher overall incidence of postoperative hematoma, ranging from 2.3% to 3.3% [47].

In our study, the observed hematoma rate is consistent with the current literature (2.5%) and hematoma is significantly more frequent in group B. This finding may be explained by the greater surgical trauma associated with thinner mastectomy flaps, the more frequent use of expander-based reconstruction, and the additional manipulation of the pectoralis major muscle. Moreover, thinner flaps are often the result of a more extensive subcutaneous dissection, which may increase the risk of injury to small perforating vessels. In addition, the reduced amount of residual subcutaneous tissue may limit the natural tamponade effect and impair local hemostasis, thereby increasing susceptibility to postoperative bleeding and hematoma formation.

Current evidence does not show jet a direct, well-established correlation in the literature between mastectomy flap thickness and the incidence of hematoma specifically in patients undergoing SNM/NSM. Nevertheless, in our series we highlight a correlation between excessive flap thinning and both hematoma formation and partial nipple–areola necrosis, suggesting the use of MRI as a useful parameter in the preoperative evaluation.

Breast MRI plays an important complementary role in the preoperative assessment of patients undergoing mastectomy, particularly in the context of SSM/NSM.

However, access to MRI for breast cancer screening or preoperative staging remains widely debated. Although MRI offers clear advantages in terms of sensitivity compared with mammography and breast ultrasound (ranging from 75 to 100%) and, according to several studies, may improve surgical planning reducing reoperation and recurrence rates, its high cost limits its widespread applicability [48–51].

Nevertheless, the results of the present study suggest that the availability of preoperative MRI imaging in patients scheduled for SSM/NSM may be useful in preventing potential complications and may also help guide the choice of reconstructive technique.

In our case series, patients with a skin flap thickness > 8 mm underwent one-stage and two-stage mastectomy in 61% and 38.9% of cases, respectively, whereas patients with a flap thickness < 8 mm underwent one- and two-stage mastectomy in 42.3% and 57.7% of cases, respectively.

These findings highlight a tendency, consistent with current recommendations, to prefer single-stage reconstruction in patients with flap adequately effective to ensure immediate prosthetic reconstruction and two-stage reconstruction in cases of thinner flaps. When analyzing the incidence of complications in our cohort, the complications occurred predominantly in cases in which these recommendations were not followed, likely due to discrepancies between preoperative and intraoperative assessments of flap thickness. These findings reinforce the concept that accurate patient selection is fundamental for successful implant-based breast reconstruction. Preoperative MRI-derived flap thickness may represent a valuable adjunct to established clinical algorithms, including the Rancati score, by providing an objective quantitative assessment of mastectomy flap thickness before surgery.

This study has some limitations. Its retrospective design and single-center setting may limit the generalizability of the findings, although similar methodological approaches have been adopted in previous studies addressing mastectomy flap assessment and reconstructive outcomes. In addition, the sample size is relatively small, mainly due to the limited availability of preoperative breast MRI in all patients undergoing surgery for breast cancer or prophylactic mastectomy in BRCA1/2 mutation carriers.

Furthermore, the inclusion of only patients who underwent preoperative breast MRI may have introduced selection bias. At our institution, breast MRI is performed only in selected clinical scenarios rather than routinely before NSM or SSM. Consequently, the study cohort may not be fully representative of the overall population undergoing these procedures, potentially limiting the generalizability of our findings.

Another limitation is the inclusion of both prophylactic and therapeutic mastectomies in the same analysis, which may represent a potential source of confounding. Patients undergoing prophylactic surgery are generally younger, have fewer comorbidities, and are less likely to receive neoadjuvant or adjuvant treatments than patients undergoing therapeutic mastectomy. These differences may have influenced the observed outcomes.

In addition, the inclusion of both one-stage implant-based reconstruction with acellular dermal matrix and two-stage reconstruction with tissue expander may have contributed to cohort heterogeneity. Although the primary predictor investigated was the preoperative MRI-derived skin flap thickness, which is not affected by postoperative tissue expansion, reconstructive technique may still influence clinical outcomes. To address this issue, we performed a subgroup analysis according to reconstructive strategy, the results of which are reported in the Appendix (Appendix 1. Table 5). However, these findings should be interpreted with caution because the study was not powered for subgroup comparisons.

**Table 5:** Univariate analysis of one stage and two stage mastectomies risk factors

	Group E (N =44)	Group F (N = 36)	P*
Age>40	36 (81.8)	32 (88.9)	0.378
BMI>30	19 (43.2)	14 (38.9)	0.698
ASA>3	0	0	n.a
Comorbidities	25 (56.8)	25 (69.4)	0.246
Smoke habits	3 (6.8)	2 (5.6)	0.816
Previous breast surgery	0	11 (30.6)	**0.001**
Previous neck/chest radiotherapy	0	3 (8.3)	0.052
NACT	4 (9.1)	4 (11.1)	0.764
Mean preoperative MRI thickness < 8 mm	10 (22.7)	12 (33.3)	0.291
*Different skin incision*			
Wise pattern	16 (36.4)	4 (11.1)	**0.009**
Periareolar	20 (45.4)	28 (77.8)	**0.003**
Inframmary	8 (18.2)	4(11.1)	0.378

Group E: one stage mastectomies; Group F: two stage mastectomies. BMI: Body mass index. ASA: American Society of Anesthesiologists. NACT: neoadjuvant chemotherapy. MRI: magnetic resonance imaging. Data are expressed as absolute values and frequencies in brackets. *Fisher’s exact test for independent samples. Percentages are expressed for each group

Another limitation is that the degree of breast ptosis was not systematically assessed or recorded in our prospective database and, therefore, could not be included in the analysis. As breast ptosis has been associated with an increased risk of ischemic complications following NSM and SSM, future studies should incorporate a standardized assessment of this variable to evaluate its combined predictive value with MRI-derived skin flap thickness.

Finally, the minimum follow-up period of three months, while adequate for evaluating early postoperative complications such as hematoma, seroma, infection, skin-flap necrosis, NAC necrosis, and early reconstructive failure, does not allow a comprehensive assessment of long-term reconstructive outcomes. Consequently, late complications, including capsular contracture, implant malposition, delayed implant loss, aesthetic outcomes, and patient-reported satisfaction, could not be adequately evaluated.

Therefore, larger prospective studies with longer follow-up periods, more homogeneous patient populations, and more detailed patient stratification are warranted to validate our findings and further define the role of MRI-derived flap thickness in guiding reconstructive decision-making after SSM/NSM.

## Conclusions

The results of this study suggest that preoperative MRI may represent a useful tool for estimating skin-flap thickness in patients undergoing SSM/NSM. Our findings indicate a potential association between MRI-assessed flap thickness and the risk of postoperative ischemic or hemorrhagic complications, which may contribute to preoperative risk stratification and support surgical planning when selecting the most appropriate reconstructive approach. However, given the limited sample size and the retrospective nature of the study, these findings should be interpreted with caution and cannot establish a definitive predictive relationship.

The ongoing debate in the literature regarding risk factors for mastectomy flap complications highlights the clinical relevance of this topic and underscores the need for larger, prospective studies with more homogeneous patient populations and detailed stratification to further investigate and validate the associations observed in the present study.

## Data Availability

The data from this study may be made available upon request to the corresponding author who will review and seek approval from the study investigators.

## Appendix

See Table 5.
